# Eco-Defense against Invasions

**DOI:** 10.1371/journal.pbio.0030429

**Published:** 2005-12-13

**Authors:** Virginia Gewin

## Abstract

Characterizing patterns of invasion across space, time, and taxonomic group will help reveal how invasive species affect ecosystem function and individual native species

Once thought only science fiction, alien invasions are one of today's major scientific challenges. The “aliens” in question are nonnative, or exotic, species capable of outcompeting natives and, ultimately, taking over the ecosystems to which they are introduced (Figure 1). Invasive alien species are a worldwide problem, now found in every ecosystem on Earth. Staving off the further advance of invasions—identified by the Millennium Ecosystem Assessment (http://www.millenniumassessment.org/en/index.aspx), a United Nations–backed audit of ecosystem health, as one of the most important drivers of ecosystem change—is a key environmental priority.

**Figure 1:**
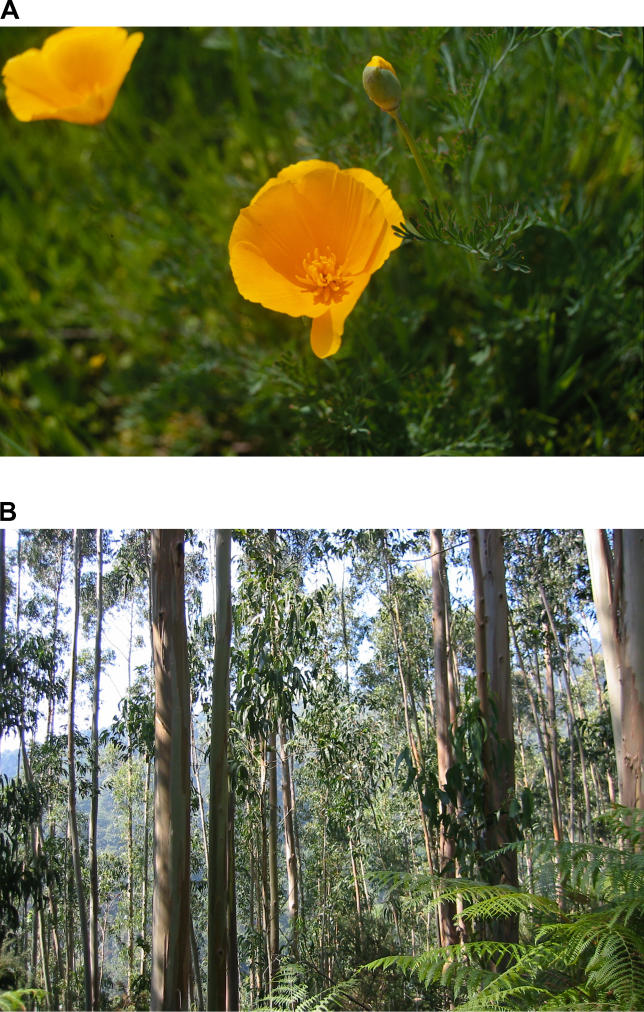
Many Species Commonly Grown throughout the World Can Become Invasive When Introduced to New Ecosystems (A) The California poppy (Eschscholzia californica) has invaded many parts of the world (e.g., Chile, Turkey, and New Zealand), and is commonly grown in many European and American gardens. (B) The blue gum tree (Eucalypus globulus)—featured here in a plantation in Spain—is one of two of the most commonly planted trees in the world, the other being the Monterey Pine. (Photos: Dov Sax)

Complicating matters, not all exotic species are invasive, or likely to cause harm to ecosystems or human health. But when introduced species exploit a specific species trait or fill a vacant niche in an ecosystem in order to invade, they cross the line from exotic to weed or pest. For example, European cheat grass (Bromus tectorum), accidentally introduced into western United States in contaminated seed, grows in the winter. Perennial grasses native to the west die off in the winter, giving cheatgrass the advantage of spreading healthy seed early in the growing season. Subsequently, Cheatgrass has altered the normal functioning of western ecosystems. Fires, once common in the region only every 60–100 years, now burn every three to four years.

Animal introductions can lead to species extinctions if they upset existing predator–prey relationships. When the poisonous brown tree snake (Boiga irregularis) was accidentally introduced to the originally snake-free island of Guam in the Pacific Ocean, as a cargo ship stowaway in 1952, it ultimately caused the demise of twelve native bird species. The ecosystem's unfilled predatory niche offered the brown tree snake the perfect opportunity to invade. Over 50 years later, the brown tree snake not only poses a human health risk (it's poisonous), but it also routinely disrupts power by crawling on electrical lines—both of which necessitate costly ongoing management.

The majority of introductions occur accidentally, as a result of human movement. From the exotic ornamental garden species to the pest control agent to the hitchhiker, these introduced nonnative species can be arbiters of economic and ecological mayhem. Governments so far have been fighting a costly, losing battle against invasions. The containment, removal, and control of invasive species costs $137 billion per year in the US alone, according to David Pimentel, economist at Cornell University in Ithaca, New York.

Conservationists say reliable reconnaissance—identifying the potential invasives early enough to implement cost-effective eradication efforts—is the best defense against invasions. But that's easier said than done. With a notorious lack of funding for this growing environmental threat, conservation managers, not surprisingly, want to focus efforts on those areas that appear most vulnerable. But even identifying those areas has proven elusive. While pragmatic, predictive approaches for identifying likely invasions must be developed, the underpinning ecological mechanisms of invasion still need to be worked out. Understanding how invasive species affect ecosystem function and individual native species will help land managers fight back. Ecologists are consequently working on several fronts to (1) identify those species most likely to be harmful, (2) resolve the spatial and temporal scale dynamics of the invasion, and (3) sharpen surveillance.

## Predictive Predicament

Despite the thousands of new exotic species introduced globally each year, only a few ever become severe problems. Carla D'Antonio, ecologist at University of California (UC), Santa Barbara, points out that many nonnatives have no impact on an ecosystem's structure or function. Once introduced, these nonnatives live among the natives without taking over their resources or crowding them out. “I'd like to see more emphasis on understanding what makes a big player and how to resolve its impact rather than simply identifying its presence,” she says. Therefore, among the many challenges in invasion biology is the development of tools to identify which exotic species are going to become invasive and where. As Jay Stachowicz, ecologist at UC Davis, puts it, “Our ability to predict invasions to date largely remains qualitative.”

Currently, prediction relies on correlative lists of known invaders and matching habitats—to create a regional “most wanted” list of likely perpetrators. But this “invasion profiling” approach isn't working, says Shahid Naeem from Columbia University in New York City. It's not simply any one unique trait, such as high reproductive rate, that allows invasives to outcompete natives as was once thought. Growing evidence suggests that an exotic species' geographic range, body size, and abundance are often similar to those of natives. But given the paucity of ecological knowledge of native species, it may not serve as a good predictor of behavior elsewhere.

“In the end an invasion is an interaction between the species' attributes and ecosystem—and the outcomes of interactions are intrinsically hard to predict,” says Mark Lonsdale, Assistant Chief of Entomology at the Commonwealth Science and Industry Research Organization in Canberra, Australia. Lonsdale, like many land managers, is skeptical of any attributes assumed capable of predicting invasibility. “A lot of people are focused on trying to find general characteristics that might distinguish invasives from noninvasives,” says Richard Duncan, ecologist at Lincoln University in Canterbury, New Zealand. “We're unlikely to find those things,” he adds. With no universal traits to search for, the task is eminently more complex. “Despite lots of desire to have a rigorous risk assessment system, there's a lot of ecology that tells us prediction is fraught with difficulty,” says Lonsdale.

But the news is not all bad, and progress is being made. David Richardson, ecologist at the Centre of Excellence for Invasion Biology in Stellenbosch, South Africa, has been successful in identifying life history attributes, from growth rate to genome size, that accurately separate invasive from noninvasive pine species. It's proved to work well for other conifers and trees in general. Indeed, many scientists believe building more general models or grouping weed species into functional groups—such as leguminous shrubs—would help determine the mechanisms behind invasions. Richardson says that researchers have been unrealistic in expecting to develop a single set of answers that would apply to all introduced species in all habitats, suggesting instead that robust rules will emerge on a regional basis.

The most unpredictable variable remains the impact of the primary mode of introduction—human activity. “Invasions are human-assisted dispersal events,” says Steven Chown, Director of Centre of Excellence for Invasion Biology. However, a proxy measure—propagule pressure, or the supply of dispersible eggs, seeds, or larvae—has emerged as an equally, if not more, important predictor of invasion success. Repeated introductions or massive movements of individuals grant a species multiple chances to escape and invade.

**Figure 2:**
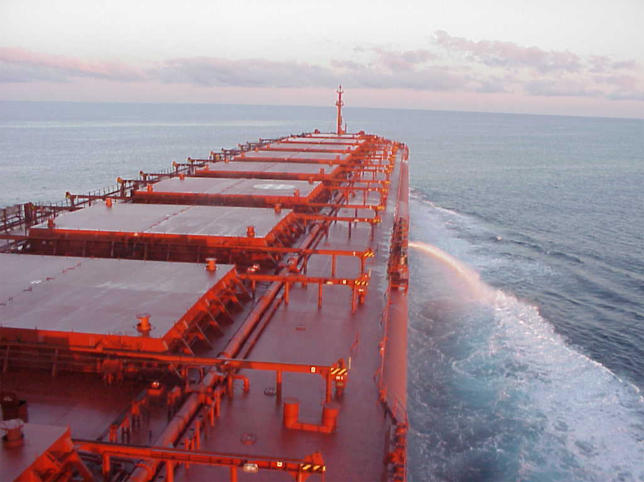
Discharging Ballast—As This Ship Is Doing off the Coast of North America—Can Transport Hundreds to Thousands of Individuals and Species at Once (Photo: Esther Collinetti)

Understanding how many organisms it takes to establish an invasive population remains a critical element of assessing risk. Propagule pressure is widely accepted as the major driver of marine coastal invasions, resulting from the dumping of a ship's ballast water (Figure 2). Discharging ballast can transport hundreds to thousands of both individuals and species at once into coastal ecosystems, but it is feasible to reduce that pressure by removing organisms via treatments such as filtration or ultraviolet radiation, thus mitigating the risk of the invasives taking hold. “It's challenging, if not impossible, to prescreen all potentially invasive marine species associated with a ship's ballast, so we must knock down the concentration of everything,” says Gregory Ruiz, invasion biologist at the Smithsonian Environmental Research Center in Edgewater, Maryland. Even more uncertain is how regional coastal influences will affect the outcome of ballast introductions. Ruiz adds that the same inoculum dumped in five different bays will have five different outcomes. “The challenge for basic ecology and management is understanding how low we need to reduce the overall density of propagules to achieve an acceptable level of risk,” says Ruiz.

Scientists are finding that no region on Earth is free from the risk of invasion, even isolated islands. We tend to think islands are easier to invade because, with fewer species, there is less competition, says Dov Sax, invasion biologist at UC Santa Barbara. But islands may have more exotic species simply because there have been so many introductions from humans drawn to go there. Even the most remote islands have been riddled with invaders (Box 1). Ironically, little work has yet been conducted in the tropical regions where most islands reside. Extrapolating findings from the better-studied temperate zones is almost certain to mask important regional mechanisms at work. For similar reasons, many are also concerned about extrapolating from one spatial scale to another.

**Box 1. Isolation: No Defense for Islands**

**Figure 3:**
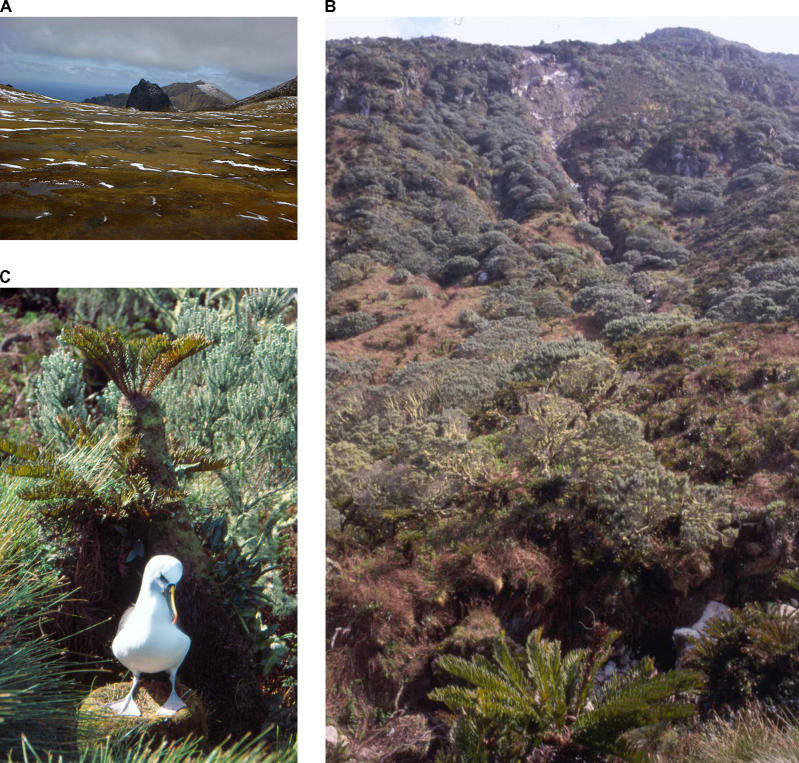
Gough Island, Remotely Located in the South Atlantic Ocean, Isn't Free from Invasive Species (A) View from the top of the island. (B) Lower down on the island, showing the tree fern Blechnum palmiforme and Phylica arborea, one of only two true trees, on the island. (C) The Atlantic yellow-nosed albatross (Thalassarche chlororhynchos), which breeds on the island, is classified as endangered (EN A4bd) on the World Conservation Union Natural Resources Red List 2004. Although originally endangered as a result of longline fishing, albatross chicks are now under threat from a mouse (Mus musculus) introduced to the island. (Photos: Steven Chown)

Gough Island in the South Atlantic Ocean is about as isolated a spot on the Earth as one can get (Figure 3). Described as one of the least disrupted ecosystems of its kind, even Gough is not free of invasion. Of the 233 recorded landings since human landfall in the 1600s, over 71 species of insects have been established through introductions. There are only 99 species of insect on the island. In other words, a new nonindigenous species is established every three to four landings. Steven Chown, Director of South Africa's Centre of Excellence for Invasion Biology, and his colleagues have calculated that the rate of human-facilitated introduction is two to three orders of magnitude greater than natural colonizations. “Alien species are getting to places never before imaginable thanks to human transport,” says Chown.

## Resolving Scale and Impact

How to scale up findings is an issue that tends to plague ecology. Invasion biology is no different. At small scales, native diversity appears to defend against invasions, but at larger scales, it is the more species-rich areas that often have the most invasions—the so-called invasion paradox. Many think that the mechanisms at work at different scales are difficult to determine because they might balance each other out. However, “although small-scale mechanisms may be masked at larger scales, it doesn't mean they are not important to understand,” says Carla D'Antonio, ecologist at UC Santa Barbara. For example, Stachowicz found that the effect of native diversity on invasion success was actually driven largely by resource availability. To tease out the possibly counteracting forces at work, a growing number of scientists are conducting experimental and observational studies at multiple scales.

In many cases, the smaller scales provide the detail necessary to define a species' preferred habitat. For example, when trying to determine the habitat requirements of two nonnative species of thistle (Carduus nutans and Carduus acanthoides), researchers found that both existed in most of the US at the state level. But on inspection of smaller-scale county data, the areas of overlap were surprisingly small between the two species. “If you had only looked at the larger spatial scale, you would have thought these two species live quite happily together,” says Katriona Shea, theoretical ecologist at Pennsylvania State University in University Park, Pennsylvania. These very similar species may have very different dispersal and distribution patterns that wouldn't have been noticed at larger scales. The greater resolution at smaller scale allows conservationists to identify risk areas for long-term monitoring and prevention.

Equally important is a better understanding of the temporal scales at which invasions bloom. “The idea that we need to not only think spatially but temporally to understand invasion pattern mechanisms has been largely ignored,” says Melinda Smith, plant community ecologist at Yale University in New Haven, Connecticut. More urgently, understanding the temporal scale is important to identify the “sleeper cells” in the environment—species that can remain apparently innocuous for years until exploding into a problem when prompted by an environmental change.

Just as intriguing are species, such as an invasive giant African land snail (Achatina fulica), that end up being empty threats. Some spontaneous population crashes of seemingly invasive species, such as this snail, are likely due to disease, but other causes of such invasive species “time lags” remain a mystery. Smith's work has shown that highly diverse communities also experience the greatest turnover of species over time, providing temporal opportunities for exotics to invade. By resolving the time scales at work for species invasions, science could help prioritize invasive species management.

While understanding the mechanisms at play at different spatial and temporal scales is important, David Lodge, ecologist at University of Notre Dame in Indiana, points out that a more important metric—and one much harder to quantify—is the impact a species has on ecosystem goods and services. “Ecologists have to be more attuned to values of society and economic costs,” he says. While one of the most studied and costly invaders, the zebra mussel (Dreissena polymorpha) in the Great Lakes region, hasn't directly caused any species extinctions, it has displaced native clams. More importantly to natural resource managers though, the mussels clog pipelines and power plants throughout the Great Lakes region. Conservative estimates put annual zebra mussel control and removal in the Great Lakes at $200 million.

## Sharpen Surveillance

Unfortunately, invasion success cannot be whittled down solely to individual attributes or profiles; effective management will depend on adequate surveillance and monitoring. The often piecemeal approach to managing invasive species, whereby different government agencies work on different invaders, is inefficient primarily because resources aren't easily shared. Effective invasive species management will require a centralized, coordinated approach linking university research to state and local agencies responsible for prevention and control efforts. It will take, says Dan Simberloff, from University of Tennessee in Knoxville, Tennessee, a national coordinating center equivalent to US Centers for Disease Control.

**Figure 4:**
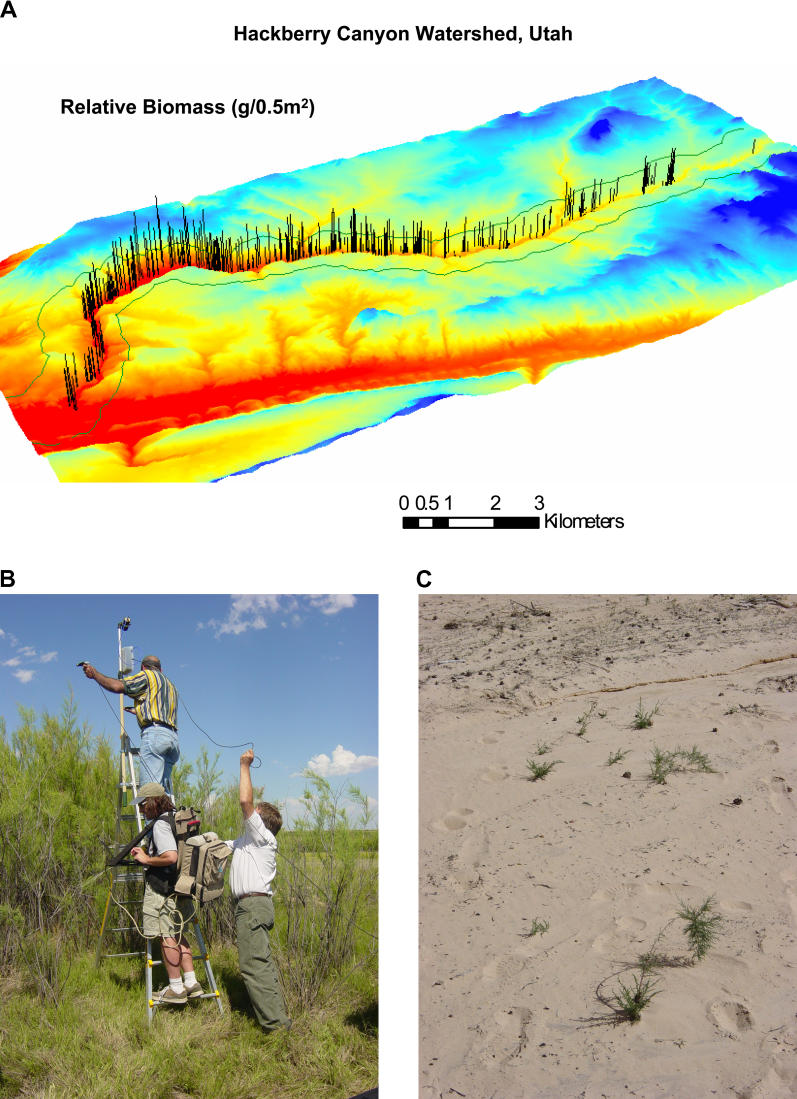
Researchers Have Teamed with NASA to Determine the Range of Potential Tamarisk Habitat (A) A computer model of Hackberry Canyon (Grand Staircase Escalante National Monument, US) that illustrates varying levels of above ground biomass of tamarisk (salt cedar) infestation. (B) Field crew using a portable spectrometer to record spectral signatures of tamarisk which will help calibrate satellite sensors. (C) Tamarisk resprouting, following a flood in Hackberry Canyon. (Figures: Paul Evangelista)

Tom Stohlgren, scientist at the US Geological Survey's Fort Collins Science Center, says the silver bullet is simply rapid online data sharing. “As scientists and agencies, we've forgotten how to share,” he says. Indeed, he's witnessed first hand the value of access to data, specifically absence data—clear indications of where the species is not found. His team partnered with NASA to develop a potential habitat model of the tamarisk (Tamarix ramosissima), a short riparian shrub also known as salt cedar (Figure 4). With data on only the presence of species, their model wasn't working in eastern US. Once they incorporated absence points from VegBank (http://www.vegbank.org) however, their model accurately predicted potential salt cedar habitat. “If we have both presence and absence data, we can get good predictive maps that are over 90% accurate,” he says. Online databases, such as that provided by the Global Invasive Species Information Network (http://www.gisinetwork.org/), are popping up as portals for sharing information.

Indeed, for birds—one of the few better-studied mobile invasive species—such long-term records of introduction success are invaluable. Often, records of bird introductions are kept so that one can determine what failed to establish and what successfully invaded. “The only way to understand characteristics of successful invaders is to have these two groups of species to compare,” says Tim Blackburn, ecologist at University of Birmingham, United Kingdom.

In lieu of meticulous historical records, scientists must use every resource available to unravel the mix of ecological forces at work. Lodge says ecologists have an important role to play in determining how to design the most efficient surveillance systems. Questions that need to be answered include the following: where should we specifically target efforts? what are reasonable detection limits? what nonnative species abundances should be of concern? when is it best to respond with eradication? and when is it more rational to simply slow the spread?

## Prospects

Every ecosystem in the world faces the threat of invasive species. Lonsdale suggests that, on average, roughly 20% of the established plant species are invasive weeds. The immediate and urgent need to prevent invasions on a global level is stimulating much-needed research. The patterns have not yet revealed all the processes at work, but the science is making strides. Most scientists now agree that it is the high biodiversity areas that are most prone to invasion—due to heavy human traffic and more favorable growth conditions—and most in need of protection. Research has also honed in on the value of understanding scale-dependent mechanisms. Moving forward, predictive tools capable of identifying potentially invasive species remain the goal. And amassing—and sharing online—the existing and future data such that it can be mined efficiently for clues is the first step.

Characterizing patterns of invasion across space, time, and taxonomic group will help dissect the ecological mechanisms at work—and none too soon. In the face of climate change, introduced species will have yet another opportunity to invade. Pinpointing species distribution limits and range shifts as the climate changes is critical to control efforts. “Understanding how ecosystems respond to exotic invasions will help us solve more applied problems, like dealing with climate change,” says Sax. Researchers in South Africa, home to many global biodiversity hot spots, are tackling this issue head on. They find that in the southern hemisphere, invasive species are weedy—grow fast and have high reproductive ability—in an environment characterized by slow growing natives. “We need to look at what predicted climate changes can do to currently present invaders and native species,” says Chown. Research efforts are underway to model potential shifts in species resulting from different climate scenarios.

Obviously, there is much yet to be learned. “The fact that so many invasions are surprisingly successful is a humbling reminder that we don't truly understand the fundamental rules of nature,” cautions Sax. A better understanding of how nature works will undoubtedly help society defend against invasions.

## Abbreviation

UC: University of California

## References

[pbio-0030429-Gaston1] Gaston K, Jones A, Hanel C, Chown S (2003). Rates of species introduced to a remote oceanic island. Proc R Soc Lond B Biol Sci.

[pbio-0030429-Kennedy1] Kennedy T, Naeem S, Howe K, Knops J, Tilman D (2002). Biodiversity as a barrier to ecological invasion. Nature.

[pbio-0030429-Levine1] Levine J, Vila M, D'Antonio C, Dukes J, Grigulis K (2003). Mechanisms underlying the impacts of exotic plant invasions. Proc Biol Sci.

[pbio-0030429-Lonsdale1] Lonsdale WM (1999). Concepts and synthesis: Global patterns of plant invasions, and the concept of invasibility. Ecology.

[pbio-0030429-Sax1] Sax D, Stachowicz J, Gaines S, Sax DF, Stachowicz JJ, Gaines SD (2005). Capstone: Where do we go from here?. Species invasions: Insights into ecology, evolution and biogeography.

[pbio-0030429-Sax2] Sax D, Brown J (2000). The paradox of invasion. Glob Ecol Biogeogr.

[pbio-0030429-Schmitz1] Schmitz D, Simberloff D (2001). Needed: A national center for biological invasions. http://www.issues.org/issues/17.4/schmitz.htm.

[pbio-0030429-Shea1] Shea K, Chesson P (2002). Community ecology theory as a framework for biological invasions. Trends Ecol Evol.

[pbio-0030429-Simberloff1] Simberloff D, Gibbons L (2004). Now you see them, now you don't—Population crashes of established introduced species. Biol Invasions.

[pbio-0030429-Stachowicz1] Stachowicz JJ, Fried H, Osman RW, Whitlatch RB (2002). Biodiversity, invasion resistance, and marine ecosystem function: Reconciling pattern and process. Ecology.

[pbio-0030429-Stohlgren1] Stohlgren T, Barnett D, Kartesz J (2003). The rich get richer: Patterns of plant invasions in the United States. Front Ecol and Environ.

[pbio-0030429-Verling1] Verling E, Ruiz GM, Smith LD, Galil B, Miller AW (2005). Supply-side invasion ecology: Characterizing propagule pressure in coastal ecosystems. Proc Biol Sci.

